# Relationships between Mitochondrial Function and Metabolic Flexibility in Type 2 Diabetes Mellitus

**DOI:** 10.1371/journal.pone.0051648

**Published:** 2013-02-13

**Authors:** Tineke van de Weijer, Lauren Marie Sparks, Esther Phielix, Ruth Carla Meex, Noud Antonius van Herpen, Matthijs Karel C. Hesselink, Patrick Schrauwen, Vera Bettina Schrauwen-Hinderling

**Affiliations:** 1 Department of Human Biology, Maastricht University Medical Center, Maastricht, The Netherlands; 2 School for Nutrition, Toxicology and Metabolism, Maastricht University Medical Center, The Netherlands; 3 Department of Human Movement Sciences, Maastricht University Medical Center, Maastricht, The Netherlands; 4 Department of Radiology, Maastricht University Medical Center, Maastricht, The Netherlands; Pennington Biomedical Research Center, United States of America

## Abstract

**Introduction:**

Mitochondrial dysfunction, lipid accumulation, insulin resistance and metabolic inflexibility have been implicated in the etiology of type 2 diabetes (T2D), yet their interrelationship remains speculative. We investigated these interrelationships in a group of T2D and obese normoglycemic control subjects.

**Methods:**

49 non-insulin dependent male T2D patients and 54 male control subjects were enrolled, and a hyperinsulinemic-euglycemic clamp and indirect calorimetry were performed. A muscle biopsy was taken and intramyocellular lipid (IMCL) was measured. *In vivo* mitochondrial function was measured by PCr recovery in 30 T2D patients and 31 control subjects.

**Results:**

Fasting NEFA levels were significantly elevated in T2D patients compared with controls, but IMCL was not different. Mitochondrial function in T2D patients was compromised by 12.5% (p<0.01). Whole body glucose disposal (WGD) was higher at baseline and lower after insulin stimulation. Metabolic flexibility (ΔRER) was lower in the type 2 diabetic patients (0.050±0.033 vs. 0.093±0.050, p<0.01). Mitochondrial function was the sole predictor of basal respiratory exchange ratio (RER) (R^2^ = 0.18, p<0.05); whereas WGD predicted both insulin-stimulated RER (R^2^ = 0.29, p<0.001) and metabolic flexibility (R^2^ = 0.40, p<0.001).

**Conclusions:**

These results indicate that defects in skeletal muscle *in vivo* mitochondrial function in type 2 diabetic patients are only reflected in basal substrate oxidation and highlight the importance of glucose disposal rate as a determinant of substrate utilization in response to insulin.

## Introduction

Multiple mechanisms have recently emerged as potential contributors to insulin resistance and type 2 diabetes progression, among them metabolic *in*flexibility, impaired mitochondrial function and intramyocellular lipid accumulation. Type 2 diabetes is characterized by both diminished rates of fatty acid oxidation in the fasting state and the inability to efficiently switch to glucose oxidation in the post-prandial state [1]. This phenomenon has been termed metabolic *in*flexibility [2], [3]. Although the importance of metabolic inflexibility in insulin resistance and type 2 diabetes has been recognized, the underlying mechanisms are poorly understood. A recent study by Galgani et al. [4] suggested that metabolic *in*flexibility is merely a reflection of insulin resistance in obesity and type 2 diabetes. The study concluded that diminished glucose disposal rate was the main determinant of blunted metabolic flexibility in individuals with type 2 diabetes. Glucose disposal rate, however, comprises two distinct parts: oxidative and non-oxidative glucose metabolism. Both of these processes are aberrantly affected in type 2 diabetes [5], [6].

Given the prominent role of skeletal muscle in substrate selection, muscle mitochondrial function is likely to have a prominent role in metabolic flexibility. However, the connection between *in vivo* mitochondrial function, metabolic flexibility and insulin resistance remains unclear.

The relationship between metabolic flexibility and insulin resistance may also be confounded by elevated plasma fatty acids and muscle lipid accumulation. These factors have been suggested to interfere with muscle insulin sensitivity but may also affect mitochondrial function or vice versa [7], [8], [9], [10], [11], [12].

Therefore, the interrelationships among mitochondrial function, metabolic flexibility and lipid metabolism in the setting of insulin resistance and type 2 diabetes warrants more investigation. In the current study, we assembled data from clamp studies performed at our research center between 2004 and 2011 to assess these interrelationships.

## Methods

### Subjects

The data in the present study is gathered from clamp studies previously performed in our research center [13], [14], [15], [16], [17]. The sample is compromised of volunteers (both diabetic and obese) who have participated in training, pharmacological or nutritional intervention studies. Here we have only used baseline data (eg. data collected prior to the intervention). Forty-nine obese non-insulin dependent sedentary male T2D patients and 54 obese sedentary male control subjects were selected. On average, controls and T2D patients had similar BMI and age. Subjects underwent a complete medical history and physical examination including history of cardiovascular, renal and pulmonary disease, cancer and duration of diabetes, routine medical laboratory tests including hematology, a maximal aerobic capacity test with concurrent ECG as previously described [18] and anthropometry. Body composition was determined using hydrostatic weighing according to Siri et al. [19]. Control subjects had no family history of T2D and underwent a 2-h oral glucose tolerance test according to World Health Organization criteria. T2D patients had well-controlled diabetes (HbA1C 7.1±0.8%) and were on monotherapy with metformin or metformin combined with sulfonylurea. All patients were diagnosed with type 2 diabetes for at least a year and the average duration of type 2 diabetes was 4.1±2.8 years. None of the subjects followed a prescribed dietary program. The Maastricht University Medical Ethical Committee approved these studies, and written informed consent was obtained on all subjects prior to screening. The clinical investigations have been conducted according to the principles expressed in the Declaration of Helsinki.

### Hyperinsulinemic-euglycemic clamp

In all subjects a 3-h hyperinsulinemic-euglycemic (40 mU /m^2^/min) clamp was performed according to DeFronzo [20]. One week prior to the clamp, subjects withdrew from their anti-diabetic medication. After an overnight fast, subjects were primed with an infusion of [6,6-2H2] glucose (0.04 mg/kg/min) to determine rates of glucose appearance (*R*a, or endogenous glucose production, EGP) and disposal (*R*d, or whole body glucose disposal, WGD) as previously described [21]. Basal and insulin-stimulated measurements were performed, including blood sampling and indirect calorimetry. Type 2 diabetic patients were clamped at slightly higher glucose values than the control subjects (5.7±0.5 mmol/L for controls vs. 6.3±1.2 mmol/L for the diabetic patients p<0.01) due to their hyperglycemia at baseline. Muscle biopsies were performed prior to the clamp under local anesthesia (2% lidocaine) according to the Bergstrom technique [22]. In these biopsies, muscle lipid accumulation was assessed histochemically in muscle cross-sections using a modified oil red O staining for fluorescence microscopy [23].

### Indirect calorimetry

During the clamp, both during the basal and the insulin stimulated state, oxygen consumption and carbon dioxide production was measured with an automated respiratory gas analyzer using a ventilated hood system (Omnical; IDEE, Maastricht, The Netherlands). The gas analyzer system was calibrated by an alcohol combustion test before every experiment. From oxygen consumption and carbondioxide production levels, whole body glucose oxidation and fat oxidation rates could be calculated using stoichiometric equations according to Frayn [24] with the assumption that protein oxidation was negligible.

### PCr-recovery by ^31^P-MRS

In a subgroup of 30 T2D patients and 31 control subjects PCr-recovery was measured by *^31^P-MRS* for *in vivo* mitochondrial function as previously described [13]. The test was performed at least 1 week prior to the clamp. A knee-extension protocol was performed on a custom-built magnetic resonance compatible ergometer with a pulley system in a 1.5-T whole-body MRI scanner (Intera; Philips Medical Systems, Best, the Netherlands). The knee extension was performed for 5 min with weight corresponding to 50–60% of the subject's pre-determined maximal knee-extension capacity. Post-exercise PCr kinetics were computed as previously described [13].

### Plasma assays

Blood collected in tubes containing EDTA was immediately centrifuged and plasma stored at −80°C until assayed. Plasma non-esterified fatty acids (NEFAs) and glucose were measured with enzymatic assays on a Cobas Fara/Mira (NEFA: Wako Nefa C test kit; Wako Chemicals, Neuss, Germany; Glucose: hexokinase method; LaRoche, Basel, Switzerland). Insulin concentration was determined using a radioimmunoassay (Linco Reseach, St. Charles, MO). Isotopic enrichment of plasma glucose was determined by electron ionization gas chromatography – mass spectrometry and expressed as tracer-to-tracee ratio.

### Calculations

Steele's single-pool non–steady-state equations were used to calculate glucose endogenous glucose production (EGP) and whole body glucose disposal (WGD) [25]. Volume of distribution was assumed to be 0.160 l/kg for glucose. Non-oxidative glucose disposal (NOGD) was calculated as WGD minus carbohydrate oxidation. Metabolic flexibility was expressed as the change in respiratory exchange ratio (ΔRER) from the fasted to the insulin-stimulated state.

### Statistics

Data are reported as means ± SE. Statistical analyses were performed using SPSS version 16.0.2 for Mac OS X (SPSS Inc., NC, USA) and JMP version 8.0 (SAS, Cary, NC, USA). Differences between groups were analyzed by one-way ANOVA. Since all variables were normally distributed, correlations were performed in a pair wise fashion using the Pearson product moment statistic. To investigate the relationships among metabolic flexibility and mitochondrial function with other metabolic characteristics, we performed stepwise linear regression analyses. Dependent variables were selected based on simple regression analyses and biological importance in the linear regression analysis. Statistical significance was set *a priori* at *p<*0.05.

## Results

### Basic characteristics

The subject characteristics are presented in Table 1. T2D patients and control subjects were comparable for body weight, BMI and age. Body composition was also similar between groups. By definition, fasting plasma glucose levels were significantly higher in T2D patients compared with control subjects (9.4±2.0 vs. 5.9±0.8 mmol/L, p<0.01); however, fasting plasma insulin levels were not different between groups. Fasting plasma NEFA levels were significantly higher in T2D patients (515.3±173.7 vs. 425.4±150.5 µmol/L; p<0.01) compared to control subjects.

**Table 1:** Subject characteristics.

	Control subjects (n)	Diabetic patients (n)	P-value
Age (years)	59.8±4.8 (54)	61.2±3.7 (49)	NS
FM (%)	30.6±6.3 (51)	30.7±5.3 (45)	NS
FFM (kg)	63.6±8.5 (51)	64.2±8.1 (45)	NS
BMI (kg/m2)	29.4±3.3 (54)	29.8±3.1 (49)	NS
VO_2_max (ml/min/kg)	31.3±6.2 (52)	27.3±5.8 (44)	<0.01
Fasted NEFA (mol/L)	425.4±150.5 (53)	515.3±173.7 (46)	<0.01
Fasted Glucose (mmol/L)	5.9±0.8 (54)	9.4±2.0 (49)	<0.01
Fasted insulin (mU/ml)	14.8±7.4 (49)	16.8±7.5 (45)	NS
HbA1c (%)	5.8±0.3 (18)	7.1±0.8 (41)	<0.01
PCr-recovery t1/2 (s)	19.8±4.5 (31)	22.3±6.9 (30)	<0.01

Basal characteristics of type 2 diabetic patients (male only) and BMI-matched controls performed in the fasting state. FM, fat mass; FFM, fat-free mass; BMI, body mass index; VO_2_max, maximal aerobic capacity; NEFA, non-esterified free fatty acids; HbA1c, glycosylated hemoglobin. In vivo mitochondrial function is described by PCr-recovery half-time rates.

### Glucose handling is impaired in type 2 diabetic patients

Oxidative and non-oxidative glucose uptake data are presented in Table 2.

**Table 2:** Hyperinsulinemic-euglycemic clamp and indirect calorimetry.

		Control subjects (n)	Diabetic patients (n)	P-value
Glucose (mmol/L)	Basal	5.7±0.5 (54)	9.0±2.1 (49)	<0.01
	During Ins. Stim.	5.3±0.4 (54)	6.3±1.2 (49)	<0.01
NEFA (µmol/L)	Basal	425.4±150.5 (53)	515.3±173.7 (46)	<0.01
	During Ins. Stim.	97.4±38.4 (54)	148.8±72.6 (49)	<0.01
	Delta	328.0±156.3 (54)	366.5±125.8 (49)	<0.01
Insulin (mU/L)	Basal	15.2±9.2 (52)	17.8±8.4 (48)	NS
	During Ins. Stim.	108.8±24.5 (52)	107.8±20.8 (48)	NS
EE (KJ/min)	Basal	5.17±0.58 (49)	5.29±0.70 (47)	NS
	During Ins. Stim.	4.66±0.86 (49)	4.77±0.95 (48)	NS
RER	Basal	0.79±0.03 (49)	0.82±0.03 (47)	<0.01
	During Ins. Stim.	0.89±0.04 (49)	0.87±0.04 (48)	<0.05
	Delta	0.093±0.05 (50)	0.050±0.033 (47)	<0.01
Glucose oxidation	Basal	6.96±2.32 (49)	8.34±2.55 (46)	<0.05
(µmol/kg/min)	During Ins. Stim.	13.69±3.74 (49)	11.97±3.25 (48)	<0.05
	Delta	6.74±3.60 (50)	3.63±2.53 (46)	<0.01
Lipid oxidation	Basal	1.09±0.24 (49)	1.01±0.22 (47)	NS
(µmol/kg/min)	During Ins. Stim.	0.55±0.25 (49)	0.72±0.22 (48)	<0.01
	Delta	0.52±0.26 (49)	0.28±0.20 (47)	<0.01
EGP (µmol/kg/min)	Basal	9.42±2.34 (54)	11.06±3.22 (48)	<0.01
	During Ins. Stim.	1.96±2.91 (54)	3.27±2.46 (49)	<0.05
	Delta	−7.45±3.96 (54)	−7.94±3.04 (48)	NS
WGD (µmol/kg/min)	Basal	9.65±2.59 (52)	11.95±3.88 (46)	<0.01
	During Ins. Stim.	28.75±9.45 (52)	20.33±7.50 (46)	<0.01
	Delta	19.86±8.58 (52)	8.37±7.42 (46)	<0.01
NOGD (µmol/kg/min)	Basal	3.19±3.16 (49)	3.62±4.56 (46)	NS
	During Ins. Stim.	15.4±8.15 (49)	8.36±6.88 (46)	<0.01
	Delta	12.29±9.12 (49)	4.74±6.80 (46)	<0.01

In the table above the results of the hyperinsulemic-euglycemic clamp and the ventilated hood measurements that were performed at baseline and during insulin stimulation are presented. Results for plasma metabolite levels are shown at baseline (before insulin stimulation) and after 3 hours of insulin infusion (during Ins. Stim.). NEFA, non-esterified free fatty acids; EE, energy expenditure; RER, respiratory exchange ratio; EGP, endogenous glucose production; WGD, whole-body glucose disposal; NOGD, non-oxidative glucose disposal.

#### Basal glucose handling

Basal whole body glucose disposal (WGD) was significantly higher in T2D patients compared to control subjects (12.0±3.9 vs. 9.4±2.3 μmol/kg/min, *p*<0.01). This was reflected in a higher fasting glucose oxidation in T2D patients (8.3±2.6 vs. 6.7±2.2 μmol/kg/min, p<0.05), whereas non-oxidative glucose disposal (NOGD) was not significantly different between groups (3.6±4.6 vs. 3.2±3.2 μmol/kg/min, p>0.05). Basal endogenous glucose production (EGP), reflecting hepatic glucose output, was higher in T2D patients compared with control subjects (11.1±3.2 vs. 9.4±2.3 μmol/kg/min; p<0.01).

#### Glucose handling during insulin stimulation

Insulin-stimulated WGD was significantly lower in T2D patients compared with control subjects (20.3±7.5 vs. 28.8±9.5 μmol/kg/min, p<0.01). This was mainly reflected in a reduced non-oxidative glucose uptake during insulin infusion in T2D patients (8.4±6.9 vs. 15.4±8.2 μmol/kg/min, *p<*0.01), although glucose oxidation during insulin infusion was also significantly lower in T2D patients (11.9±3.3 vs. 13.7±3.74 µmol/kg/min, p<0.05). EGP was almost completely suppressed during insulin infusion in both groups, although T2D patients still had significantly higher EGP during insulin stimulation (3.3±2.5 vs. 1.9±2.9 µmol/kg/min, p<0.05).

### Type 2 diabetic patients have a blunted insulin suppression of lipid oxidation compared to controls despite similar IMCL content

Data on fatty acid handling fasting and during the clamp are presented in Table 2.

#### Basal lipid handling

As mentioned above, plasma NEFA levels in the fasted state were significantly higher in T2D patients (Table 1, p<0.01). Interestingly, intramyocellular lipid content (IMCL) was not significantly different between the two groups (1.10±0.64 vs. 0.96±0.66% for T2D patients and control subjects, respectively, p = 0.52). In the fasted state, basal lipid oxidation was the same in both groups (1.0±0.2 and 1.1±0.2 for T2D patients and controls, respectively; p>0.05).

#### Lipid handling during insulin stimulation

Although plasma NEFA levels decreased upon insulin stimulation in both groups, they remained significantly elevated in the T2D patients compared to the controls (148.8±72.6 vs. 97.4±38.4 µmol/L, p<0.01). Insulin stimulation significantly decreased lipid oxidation in both groups, although to a lesser extent in the T2D patients compared to controls (−0.3±0.2 vs. −0.5±0.3 μmol/kg/min, p<0.05).

EE did not significantly change upon insulin stimulation in the type 2 diabetic patients nor in the obese controls. Basal EE also did not differ between the groups (see table 2).

### Type 2 diabetic patients are metabolically inflexible compared with controls

Respiratory exchange ratios in basal and insulin-stimulated states are presented in Table 2. Fasting respiratory exchange ratio (RER) was significantly higher in the T2D patients (0.82±0.03 vs. 0.79±0.03, p<0.01) and significantly lower upon insulin stimulation (0.87±0.04 vs. 0.89±0.04, p<0.05) compared with age-and BMI-matched controls. This resulted in a significantly decreased metabolic flexibility (ΔRER), i.e. a lower increase in RER from fasting to insulin-stimulated state (0.050±0.033 vs. 0.093±0.050, p<0.01).

### Impaired in vivo mitochondrial function in type 2 diabetic patients versus age- and BMI-matched controls

Mean PCr recovery half-time (PCr-t1/2) was prolonged by 12.5% in T2D patients compared with control subjects (22.3±6.9 vs. 19.8±4.5 seconds, respectively; p<0.01, Table 1), thus indicating impaired *in vivo* mitochondrial function in T2D patients. None of the subjects showed significant acidification at the end of the exercise protocol, and the pH decreased similarly from rest to end-exercise in both groups (end-exercise pH: 7.05±0.02 and 7.02±0.03, p>0.05; delta-pH 0.07±0.02 and 0.08±0.03, p>0.05, in T2D patients and control subjects, respectively; data not shown).

### Simple correlations reveal that mitochondrial function and glucose disposal rate are determinants of metabolic flexibility

To assess the relationship between the distinct parameters, we performed a simple bivariate correlation analysis. This analysis revealed that metabolic flexibility was positively associated with WGD during insulin stimulation (R^2^ = 0.17, p<0.01) and inversely related to *in vivo* PCr-recovery half-time (PCr-t_1/2_, R^2^ = 0.07, p<0.05). PCr-recovery rates were not significantly associated with IMCL (data not shown), but they were inversely related to WGD during insulin stimulation in all subjects (R^2^ = 0.10, p<0.05). This effect seemed to be mainly influenced by non-oxidative glucose disposal, as this, in contrast to glucose oxidation, negatively correlated with PCr-t_1/2_ (R^2^ = 0.07, p<0.05).

### Rate of glucose disappearance is a good predictor of metabolic flexibility in both type 2 diabetic patients and BMI-matched controls

**Figure 1:**
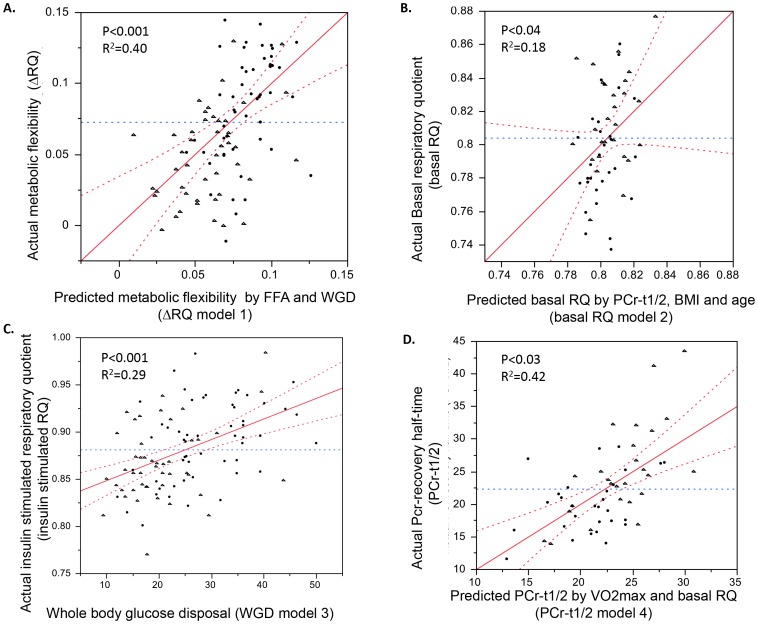
Predicted relationships from stepwise linear regression analyses; on the x-axis you can find the values predicted by the models by the different parameters in the model, on the y-axis you can find the actual value as measured in the study. The results were plotted for the following models: (**A**) Model 1, relationship between actual metabolic flexibility (ΔRER) and the metabolic flexibility predicted by Model 1 (by plasma NEFA and WGD during insulin stimulation), (**B**) Model 2; relationship between actual basal respiratory exchange ratio (basal RER) and the basal RER predicted by Model 2 (by plasma PCr-recovery half-time, BMI and age), (**C**) Model 3; relationship between insulin-stimulated respiratory exchange ratio (ins. stim. RER) and the insulin-stimulated RER predicted by Model 3 (by WGD during insulin stimulation), (**D**) Model 4; relationship between actual PCr-recovery half-time and PCr-recovery half-time predicted by Model 4 (by basal RER and VO2max).

To further investigate, whether these simple correlations were not confounded by other parameters, we performed a stepwise linear regression analyses to identify the main factors that predict metabolic flexibility (Table 3). Similar to the findings of Galgani et al.[4], insulin-stimulated plasma NEFA levels and whole body glucose disposal rate significantly contributed to the model (R^2^ = 0.40, p<0.001; Model 1, Table 3 & Figure 1A). Whilst PCr-recovery half-time (i.e. *in vivo* mitochondrial function) was related to metabolic flexibility in simple bivariate correlation analysis, it was not a significant predictor of metabolic flexibility (Model 1, Table 3 & Figure 1A).

**Table 3:** Stepwise Linear regression analyses.

Model	Dependent variable	Parameters	Significant Predictors	Beta	R^2^	P-value
1	Metabolic flexibility (delta RER)	NEFA clamp WGD clamp EE clamp T1/2 PCr HbA1c BMI Age VO2max	NEFA clamp WGD clamp	−0.252 0.304	0.40	<0.001
2	RER basal	NEFA basal WGD basal EE basal T1/2 PCr HbA1c BMI Age VO2max	T1/2 PCr Age BMI	−0.284 −0.186 −0.221	0.18	<0.04
3	RER insulin stimulated	NEFA clamp WGD clamp EE clamp T1/2 PCr HbA1c BMI Age VO2max	WGD	0.457	0.29	<0.001
4	T1/2 PCr	VO2max T2D Age BMI	VO2max T2D	−0.516 0.224	0.401	<0.01

In the above table the results of the stepwise regression analyses are presented. Indicated in the “Parameters” column are all variables included in the model, and in the “Significant Predictors” column are all variables that were significant after stepwise regression. In the Beta column, the beta value for each (significant) predictor in the model is given and in “R^2^” column are the R^2^ values for the entire model. P<0.05 was considered statistically significant.

### The individual components of metabolic flexibility (basal and insulin-stimulated respiratory exchange ratio) are defined by different metabolic parameters

Next, we sought to determine the predictors of the individual components of metabolic flexibility, fasting RER under basal conditions and insulin-stimulated RER. Interestingly, *in vivo* mitochondrial function, determined by PCr-recovery half-time, was the only significant predictor of basal RER (R^2^ = 0.18, p<0.04; Model 2, Table 3 & Figure 1B). As expected, only whole body glucose disposal rate during insulin stimulation (WGD) was a significant predictor of insulin-stimulated RER (R^2^ = 0.29, p<0.001; Model 3, Table 3 & Figure 1C). Plasma NEFA levels did not significantly contribute to the prediction of insulin-stimulated RER.

## Discussion

In our present study, we confirm that type 2 diabetic patients are metabolically inflexible and have reduced *in vivo* mitochondrial function. Controversy exists regarding the relationship between mitochondrial function and type 2 diabetes.

In this study *in vivo* mitochondrial function was measured by assessing the half-time of PCr resynthesis after exercise with ^31^P-MRS. The PCr-recovery kinetics reflect the capacity to oxidatively generate ATP, and depends among others on intrinsic mitochondrial function, mitochondrial density and perfusion, thereby reflecting muscle oxidative capacity. *Ex vivo* assays, like high resolution respirometry, mitochondrial density assays and measurements of ROS production more accurately assess the sources of the decreased capacity. To evaluate to what extent mitochondrial function is determining metabolic flexibility, a measurement of the *in vivo* situation is warranted.

It has been speculated that mitochondrial abnormalities drive an impaired lipid oxidation with augmented lipid storage in skeletal muscle as a consequence. This lipid storage and defects in beta-oxidation have been shown to parallel a decrease in insulin sensitivity and metabolic flexibility via impairment of insulin signaling pathways and a subsequent decline in glucose uptake by lipotoxic intermediates [26], [27], [28], [29], [30], [31]. Indeed we did find an impaired mitochondrial capacity in our type 2 diabetic patients. However, the reduction in insulin sensitivity and mitochondrial function as observed in our type 2 diabetic patients was independent of intramyocellular lipid content, which was similar between the two groups. Although this could suggest that intramyocellular lipid storage may not be a main determinant of insulin sensitivity when BMI and age matched obese and T2D patients are compared, we cannot rule out the possibility that lipid intermediates were different between groups. Also we did not do any *ex vivo* assays to more accurately assess the source of the decreased capacity. Hence several questions remain which can not be answered by the correlative analysis performed in this study.

However, we did find a relationship between in vivo mitochondrial capacity and metabolic flexibility. The relationship of mitochondrial capacity with metabolic flexibility disappeared when we added whole body glucose disposal to the model. Upon a more thorough analysis of the different components of metabolic flexibility, we found that *in vivo* mitochondrial function was the single predictor of basal RER. In contrast, insulin-stimulated RER was mainly determined by glucose disposal rate and not by *in vivo* mitochondrial function. This suggests that reduced mitochondrial function does not negatively impact the ability of the skeletal muscle to switch substrates; rather it is primarily responsible for basal substrate utilization. Moreover, substrate oxidation during insulin stimulation seems to be mainly determined by substrate availability, such as insulin-stimulated glucose uptake, which is in agreement with the work by Galgani and colleagues [4].

Consistent with the finding that insulin-stimulated RER is mainly dependent on glucose disposal rates, Kelley et al. [2] demonstrated that in obese patients after weight loss, both insulin sensitivity and insulin-stimulated RER normalized to the level of their lean controls. While it is well known that weight loss does not improve mitochondrial function [32], [33], in the study of Kelly et al, Baseline RER could not be restored after weight loss. This might fit with our theory that basal RER is mainly dependent on mitochondrial function.

Besides glucose disposal rates, also plasma NEFA levels, seemed an important determinant of metabolic flexibility. Both whole body glucose disposal and plasma NEFA levels during insulin stimulation significantly contributed to the prediction of metabolic flexibility, which also supports the findings of Galgani et al [4]. Plasma free fatty acid levels were positively associated with fatty acid oxidation rates (both in the fasted (R^2^ = 0.047, p<0.05) and in the insulin-stimulated state (R^2^ = 0.047, p<0.05)); therefore it might be plausible that the influence on metabolic flexibility is primarily substrate driven, as proposed by the Randle cycle theory [28].

It might be speculated that reduced mitochondrial function in diabetic patients is influenced by differences in physical activity levels between the groups [34], [35]. Indeed, VO2max (reflecting physical fitness) is reduced in the diabetic patients. We addressed this issue by correcting for maximal aerobic capacity (VO2max) and found that the difference in *in vivo* mitochondrial function remained significantly reduced in the type 2 diabetic patients compared to their controls (data not shown). This suggests that this defect is more than just a difference in training status in these patients. Even so, one can question why *in vivo* mitochondrial function measured by PCr recovery – reflecting ATP synthetic capacity – would affect basal substrate oxidation. Thus, a reduced capacity to synthesize ATP could be due to increased mitochondrial uncoupling, which might be compensated by increased oxidation in the basal state. However, it should be noted that uncoupling only accounts for a small part of the total oxidation rates in skeletal muscle, and as energy expenditure was not significantly different, the effect of muscle mitochondrial uncoupling on the whole body oxidation rates in this study probably is small. Actually, if anything, earlier results indicated that mitochondrial uncoupling would be decreased in diabetes rather than increased [31], [36], [37], [38], [39]. However, more studies are needed to understand how mitochondrial function can affect basal substrate oxidation.

In conclusion, the current study advances the concept that mitochondrial function is a main determinant of basal substrate handling, whereas glucose disposal rate is the primary determinant of insulin-stimulated substrate handling. Furthermore, plasma free fatty acid levels are significantly elevated in type 2 diabetes when compared to obese subjects with similar age, BMI and fat mass, and may contribute to the defects consistently observed in metabolic flexibility in individuals with type 2 diabetes.
